# Relationship between high-fat diet, gut microbiota, and precocious puberty: mechanisms and implications

**DOI:** 10.3389/fmicb.2025.1564902

**Published:** 2025-06-04

**Authors:** Nan Wu, Ke Ning, Yanan Liu, Qinghua Wang, Ning Li, Lei Zhang

**Affiliations:** ^1^Microbiome-X, School of Public Health, Cheeloo College of Medicine, Shandong University, Jinan, China; ^2^Jinan Institute of Child Health Care, Children’s Hospital Affiliated to Shandong University (Jinan Children’s Hospital), Jinan, China; ^3^School of Biological Science and Technology, University of Jinan, Jinan, China

**Keywords:** high-fat diet, gut microbiota, precocious puberty, probiotics, gut metabolites

## Abstract

Precocious puberty (PP) is the second most common pediatric endocrine disorder globally and poses a growing public health concern, particularly among girls. While the exact biological mechanisms underlying PP remain unclear, unhealthy dietary patterns, particularly the consumption of a high-fat diet (HFD), are recognized as significant modifiable risk factors. The gut microbiota (GM) is an environmental factor that is disrupted by HFD and may modulate the onset and progression of PP. This review explored the intricate relationship between HFD, GM, and PP, and elucidated the potential mechanisms by which HFD may promote PP development by summarizing evidence from preclinical to clinical research, focusing on the role of GM and its derived metabolites, including short-chain fatty acids, bile acids, lipopolysaccharides, and neurotransmitters. Mechanistic exploration provides novel insights for developing microbiota-targeted therapeutic strategies, such as dietary and lifestyle interventions, fecal microbiota transplantation, probiotics, and traditional Chinese medicine, paving the way for promising approaches to prevent and manage PP.

## 1 Introduction

Precocious puberty (PP) is characterized by the premature emergence of secondary sexual characteristics—before age 8 in girls and 9 in boys (Alghamdi and Alghamdi, 2023). PP can be classified into two main categories considering the underlying pathogenetic mechanism: central PP (CPP) and peripheral PP (PPP). CPP is caused by early activation of the hypothalamic–pituitary–gonadal (HPG) axis, leading to the development of both primary and secondary sexual characteristics. This form accounts for approximately 80% of PP cases. In contrast, PPP results from factors that elevate steroid hormone levels to those typical of puberty, independent of gonadotropin secretion (For detailed discussions on the etiology, diagnosis, and treatment of both subtypes, see the review; Cheuiche et al., 2021). Notably, the incidence of PP in girls is markedly higher than in boys, with a rate 15 to 20 times greater, and its global prevalence continues to rise (Shim et al., 2021; Park et al., 2021). The onset of PP results from a complex interaction of genetic, dietary, environmental, and lifestyle factors. Among these, a high-fat diet (HFD) has emerged as a modifiable risk factor that has garnered significant attention.

With advancements in microbiomics, the gut microbiota (GM) and its derived metabolites have been increasingly recognized as important molecules in the gut-organ crosstalk, exerting either beneficial or detrimental effects on various extra-intestinal organs. Mounting evidence indicates various perturbations in GM associated with PP. Notably, excessive fat consumption in HFD results in consequences such as gut dysbiosis, gut barrier dysfunction, emphasizing GM’s potential mediating role in the pathogenesis of PP induced by HFD. In this review, we summarized studies exploring the relationship between HFD, GM, and PP and specifically discussed the direct effects of HFD on PP and its indirect effects mediated by GM and its metabolites. Finally, we reviewed the potential of GM regulation as a strategy to mitigate or manage PP, offering valuable insights for future research and therapeutic interventions.

## 2 HFD promotes PP

HFD significantly disrupts neuroendocrine and metabolic homeostasis, serving as a critical environmental determinant in the pathogenesis of PP. Robust epidemiological studies have revealed differential effects of HFD components on pubertal timing (Supplementary Table 1; Chen et al., 2024; Gu et al., 2024; Günther et al., 2010; Xiong et al., 2022; Du et al., 2024; Cheng et al., 2021b; Xu et al., 2022; Cheng et al., 2021a; Shahatah et al., 2021; Nguyen et al., 2020; Jansen et al., 2016; Mueller et al., 2015; Carwile et al., 2015; Mervish et al., 2013; Wiley, 2011; Rogers et al., 2010; Koo et al., 2002; Berkey et al., 2000; Maclure et al., 1991; Merzenich et al., 1993; Szamreta et al., 2020). Animal protein intake is consistently associated with accelerated pubertal development, manifesting as premature onset of thelarche, enhanced growth velocity, and earlier menarche (Gu et al., 2024; Günther et al., 2010; Cheng et al., 2021b; Rogers et al., 2010; Berkey et al., 2000). Conversely, milk’s effects remain controversial, with studies reporting negative (Du et al., 2024; Wiley, 2011), positive (Wiley, 2011), or no significant associations (Carwile et al., 2015) with early pubertal onset, potentially due to genetic backgrounds, study design, dietary intake assessment, and temporal context. These inconsistencies highlight the need for future studies to control confounding variables more rigorously to derive precise conclusions.

Regarding dietary lipids, polyunsaturated fatty acids (PUFAs)—essential steroidogenesis precursors—exhibited a dose-dependent positive correlation with earlier pubertal onset and menarche in girls (Xu et al., 2022; Cheng et al., 2021a; Nguyen et al., 2020; Rogers et al., 2010), whereas monounsaturated fatty acids (MUFAs) demonstrated inhibitory effects (Nguyen et al., 2020). Additionally, plant-derived components such as flavonoids, soy, and dietary fiber were associated with delayed pubertal timing (Xiong et al., 2022; Nguyen et al., 2020; Mervish et al., 2013), suggesting potential preventive strategies through reduced animal protein/dietary fat consumption and increased plant-based diet intake.

**Figure 1:**
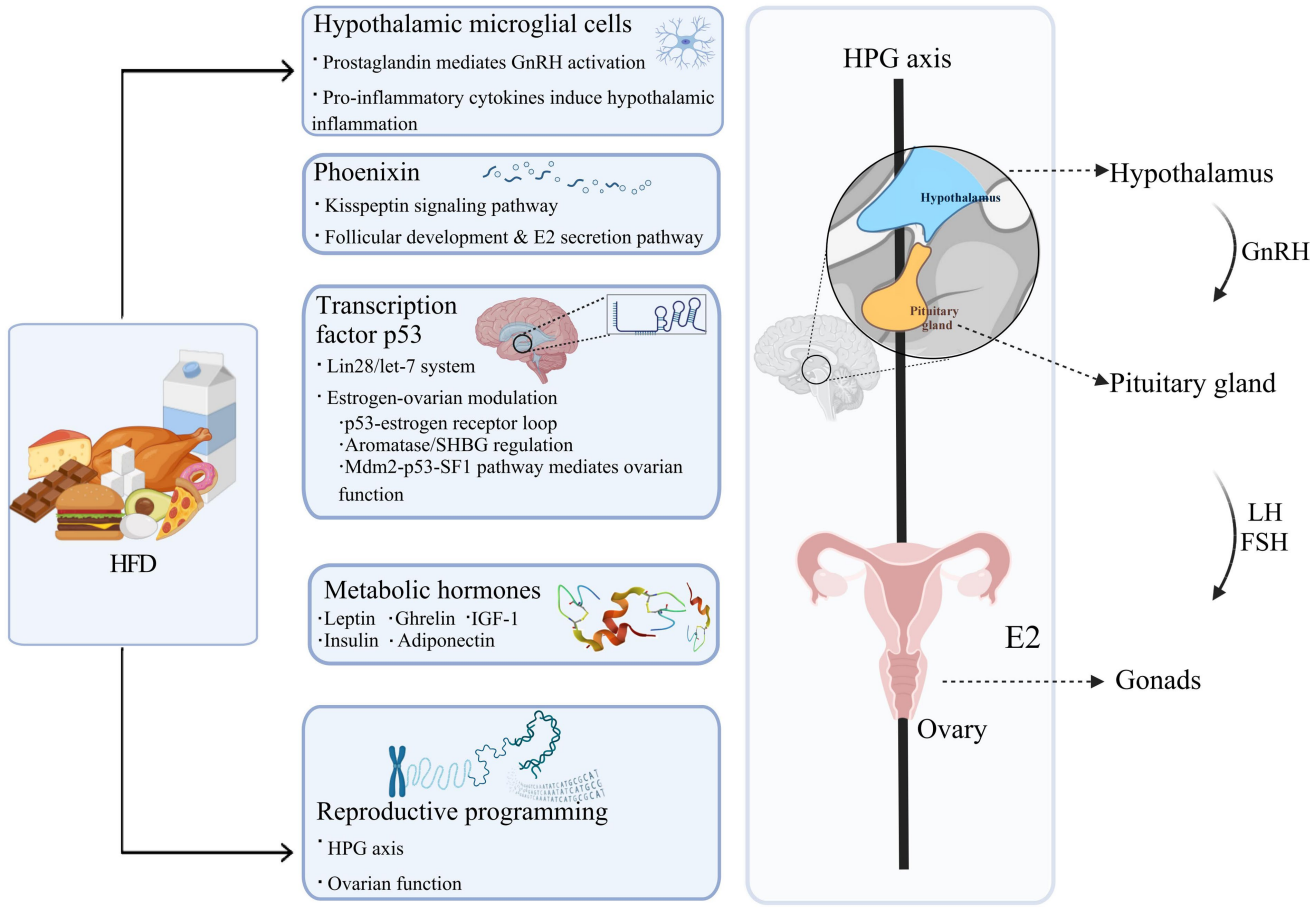
The pathways through which HFD contributes to PP. HFD, a high-fat diet; GnRH, gonadotropin-releasing hormone; LH, luteinizing hormone; FSH, follicle-stimulating hormone; HPG axis, hypothalamic–pituitary-gonadal axis; E2, estradiol; SHBG, sex hormone-binding globulin; Mdm2, mouse double minute 2 homolog; SF1, steroidogenic factor 1; IGF-1, insulin-like growth factor-1. Figure created with BioRender.com.

Human studies primarily provided observational evidence, but preclinical research established direct causality, demonstrating that maternal or female offspring exposure to HFD significantly accelerated vaginal opening (VO), a key marker of puberty onset, and gonadal maturation (Bo et al., 2022; Wang et al., 2020). The underlying mechanisms are illustrated in Figure 1.

### 2.1 Hypothalamic microglial cells

Microglial cells play a critical role in the context of PP and exhibit heightened sensitivity to fatty acids, particularly long-chain saturated fatty acids, which further enhance their activation (Wang et al., 2012; Gupta et al., 2012). Activated hypothalamic microglia release prostaglandins, acting as neurotrophic factors that stimulate GnRH neurons and subsequently hasten puberty (Sheremeta et al., 2024; Fujioka et al., 2013). Additionally, sustained HFD exposure further activates microglial cells to secrete pro-inflammatory cytokines, including interleukin-1β, interleukin-6, nitric oxide (NO), tumor necrosis factor-*α*, and reactive oxygen species, which collectively induce hypothalamic inflammation and exacerbate the aberrant activation of GnRH neurons (Tzounakou et al., 2024; Chen et al., 2022). This mechanism has been thoroughly reviewed by Stathori et al., whose comprehensive analysis elucidates the potential pathways through which HFD-induced neuroinflammation affects the activity of HPG axis (Stathori et al., 2025). Intriguingly, evidence suggests that HFD Short-term HFD induces triggers microglial activation beyond the effects of obesity, suggesting that HFD introduces unique stressors to the central nervous system, amplifying microglial activation (Gao et al., 2014).

### 2.2 Phoenixin

Phoenixin, a reproductive peptide crucial for regulating the estrous cycle, is elevated in response to fatty acids such as palmitate and oleate (Mcilwraith et al., 2018), thereby accelerating puberty through GnRH activation mediated by kisspeptin (Stein et al., 2016; Gozukara et al., 2016; Dhillo et al., 2005; Clarke and Dhillo, 2016). In addition, research studies have demonstrated that Phoenixin not only impairs the HPG axis but also adversely affects the follicular development and function. Nguyen et al. were the first to identify Phoenixin, along with its receptor G-protein coupled receptor (GPR) 173, in the ovarian follicles of women post-cervical cancer surgery. *In vitro* studies further verified Phoenixin’s capacity to promote follicular growth and increase ovulated oocyte numbers, as well as enhance estradiol (E2) secretion in a dose-responsive manner (Nguyen et al., 2019; Clarke and Dhillo, 2019). Overall, these findings establish Phoenixin serves as a key mediator of HFD-induced PP by integrating metabolic signals with the neuroendocrine system and ovarian development.

### 2.3 Transcription factor p53

The transcription factor p53, a well-known tumor suppressor, has also been found to regulate the genetic pathways governing puberty onset (Chen et al., 2020). In the HFD-induced PP model, hypothalamic p53 expression positively correlated with pubertal onset timing, where p53 overexpression significantly accelerated puberty progression, providing causal evidence for p53 in the development of PP (Chen et al., 2021). Mechanistically, p53 exerts dual regulatory effects through the Lin28/let-7 system: centrally, it activates the Kiss1/GPR54 and phosphatidylinositol 3-kinase (PI3K)-mammalian target of rapamycin (mTOR) signaling pathways to stimulate GnRH secretion (Chen et al., 2021; Xie et al., 2025); gonadally, it directly promotes ovarian granulosa cells proliferation and E2 synthesis (Sui et al., 2023). In addition, p53 orchestrates estrogen signaling and ovarian function through multiple mechanisms: forming a bidirectional regulatory loop with estrogen receptor *α* (ERα) that enables mutual transcriptional control through promoter binding and estrogen response element-mediated transactivation (Shirley et al., 2009; Berger et al., 2012); modulating estrogen levels and bioactivity by regulating the expression of aromatase (the rate-limiting enzyme in estrogen synthesis) and sex hormone-binding globulin (SHBG; Charni-Natan et al., 2019); and participating in ovarian function regulation via the mouse double minute 2 homolog (Mdm2)-p53-steroidogenic factor 1(SF1) signaling pathway in granulosa cells (Zanjirband et al., 2023). While our understanding of the mechanisms and regulation of p53 in HFD-induced PP is gradually improving, the upstream mechanisms by which HFD upregulates p53 expression are not completely understood. However, emerging evidence suggests that ketone bodies, particularly *β*-hydroxybutyrate, may play a potential role in this regulatory process (Roberts et al., 2017).

### 2.4 Metabolic hormones

Long-term HFD consumption disrupts the regulation of key metabolic hormones, which regulate both energy homeostasis and the pathogenesis of PP. Clinical observations reveal that girls with PP exhibit characteristic endocrine alterations, including elevated leptin (Zurita-Cruz et al., 2021), insulin (Zhang et al., 2025), and insulin-like growth factor-1 (IGF-1; Zhang et al., 2025) levels alongside reduced adiponectin (APN; Zurita-Cruz et al., 2021; Sitticharoon et al., 2017), while ghrelin changes remain inconsistent (Tarçin et al., 2024; Zhu et al., 2008). Mechanistic investigations have elucidated distinct neuroendocrine pathways through which metabolic hormones coordinate pubertal timing. Leptin accelerates sexual maturation through dual mechanisms, including central activation of hypothalamic Kisspeptin-GnRH neurons and direct stimulation of ovarian granulosa cell function (Childs et al., 2021). APN, conversely, exerts inhibitory control by suppressing GnRH neuronal activity through adenosine-monophosphate-activated protein kinase (AMPK)-mediated pathway (Mathew et al., 2018). Ghrelin activates GnRH neurons via growth hormone secretagogue receptors (GHSR) or kisspeptin-dependent pathways, and potentially modulates the HPG axis through adrenocorticotropic hormone (ACTH; Farkas et al., 2013; Shi et al., 2022). Insulin enhances leptin synthesis to exert synergistic effects, while IGF-1 likely regulates pubertal progression through multiple mechanisms involving IGF-binding proteins, GnRH neurons, Kisspeptin neurons, and gonadotropins (Salvi et al., 2006).

Collectively, these hormones act on both the hypothalamus and ovarian tissues to regulate pubertal progression, with leptin, insulin, and IGF-1 primarily exerting stimulatory effects while APN and ghrelin mainly demonstrate inhibitory actions.

### 2.5 Reproductive programming

Reproductive system development begins around the fifth week of gestation and continues until after birth, during which newborns experience a temporary surge in gonadotropin levels, triggering a phenomenon known as “mini-puberty.” Following this transient activation, the HPG axis becomes dormant through a combination of gonadal and non-gonadal inhibitory mechanisms (Yao et al., 2021). These physiological changes highlight how adverse environmental conditions during pregnancy and the early postnatal period can significantly affect the development and function of the reproductive system.

Maternal overnutrition or obesity during pregnancy and lactation substantially accelerates reproductive maturation in offspring have been demonstrated. A large-scale cohort study found that daughters of mothers who gained more than 40 pounds during pregnancy were 30% more likely to experience menarche before age 11 (Boynton-Jarrett et al., 2011). Similarly, female offspring of maternal mice fed HFD during pregnancy and lactation also exhibited earlier puberty onset, with increased E2 levels and decreased luteinizing hormone (LH) levels. Interestingly, supplementation with conjugated linoleic acid during these stages effectively reversed early-onset puberty in offspring (Reynolds et al., 2015). Collectively, maternal HFD during pregnancy and lactation promotes early puberty and reproductive system abnormalities in offspring by disrupting the developmental programming of the HPG axis and ovarian function, with dietary fatty acids potentially influencing this process.

## 3 HFD disrupts the balance of the GM

The GM exhibits high sensitivity to dietary shifts and changes in the physiological state of the digestive system, with effects observable within 24 h (Qin et al., 2020). Short-term HFD exposure induces dysbiosis characterized by an elevated Firmicutes/Bacteroidetes ratio (Shang et al., 2017), concomitant with increased abundance of bile-resistant genera (e.g., *Alistipes*, *Bacteroides*) and decreased populations of plant polysaccharide-degrading Firmicutes (e.g., *Roseburia*, *Ruminococcus bromii*; Bisanz et al., 2019; David et al., 2014). Importantly, while short-term HFD induces transient microbial shifts, long-term dietary patterns exert more profound effects: European children consuming a long-term HFD, rich in animal proteins and fats, exhibited a gut microbiome predominantly composed of *Bacteroides* enterotypes, contrasting sharply with the microbial profiles of Burkinabe children maintained on traditional carbohydrate-rich, low-protein diets (De Filippo et al., 2010; Daniel et al., 2014; Wu et al., 2011).

The types of fatty acids prevalent in HFD also have important consequences for both GM and health. Elevated n-6 PUFA levels promote the proliferation of pro-inflammatory species, such as *Mucispirillum schaedleri* and *Lactobacillus murinus* (Selmin et al., 2021), establishing a pro-inflammatory intestinal milieu that exacerbates metabolic endotoxemia and harms health. Furthermore, increased intake of saturated fatty acids, another key component of HFD, facilitates the entry of Gram-negative bacterial lipopolysaccharides (LPS) into the bloodstream, triggering the release of pro-inflammatory cytokines and activating the toll-like receptor 4 signaling pathway, contributing to insulin resistance and inflammation (Hu et al., 2022).

## 4 Gut microbial imbalance promotes PP

Evidence that antibiotic exposure may elevate the risk of PP in children points toward a role of gut microbiome dysbiosis in PP pathogenesis (Hu et al., 2022). Multiple 16S rRNA gene sequencing studies have revealed global perturbations in the GM of girls with PP compared to healthy controls (Supplementary Table 2; Bo et al., 2022; Wang et al., 2020; Wang Y. et al., 2024; Wang L. et al., 2024; Huang et al., 2023; Huang et al., 2022; Li et al., 2021b; Dong et al., 2020; Qian et al., 2024; Yi et al., 2024; Nguyen et al., 2024; Yuan et al., 2023; Wang et al., 2022), characterized by altered abundances of key taxa (such as *Ruminococcus*; Wang Y. et al., 2024; Huang et al., 2022; Dong et al., 2020), *Bifidobacterium* (Wang L. et al., 2024; Huang et al., 2023; Qian et al., 2024), and *Bacteroides* (Wang Y. et al., 2024; Wang L. et al., 2024; Huang et al., 2022; Qian et al., 2024), along with increased butyrate-producing bacteria (Wang Y. et al., 2024; Huang et al., 2022), and enhanced metabolic potential for neuroendocrine and oxidative stress-related pathways (Wang Y. et al., 2024; Huang et al., 2023; Li et al., 2021b). These findings were further validated in PP animal models (Bo et al., 2022; Yi et al., 2024). Integrating evidence on microbiota-derived metabolites such as short-chain fatty acids (SCFAs) and bile acids (BAs; detailed in Section 5.2), these observations suggest a role for GM and their metabolites in PP development.

However, cross-sectional studies cannot distinguish whether microbial alterations represent causative “driver” microorganisms or secondary “passenger” species proliferating in the PP microenvironment. To address this limitation, recent animal experiments utilizing fecal microbiota transplantation (FMT) from PP donors (either human patients or PP model rats) to recipient female rats demonstrated accelerated puberty onset, accompanied by elevated serum levels of LH, follicle-stimulating hormone (FSH), and E2, as well as upregulated hypothalamic *Kiss1* and *Gnrh* gene expression (Bo et al., 2022; Qian et al., 2024). These findings provided compelling evidence that GM can promote PP through modulation of the HPG axis, although the precise molecular mechanisms by which specific microbial taxa and their metabolic products regulate pubertal timing remain to be elucidated.

## 5 HFD promotes PP development through changes in GM

**Figure 2:**
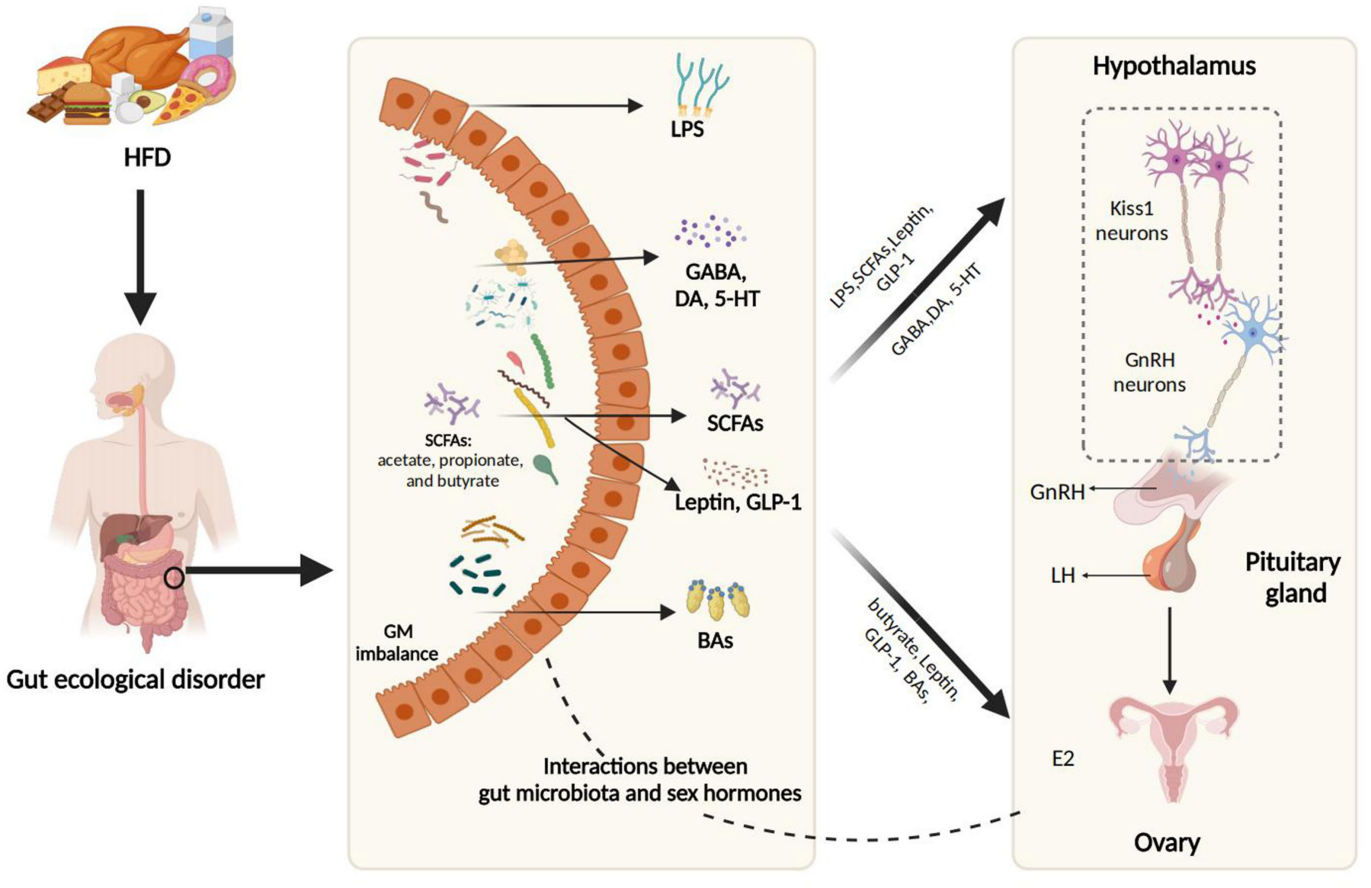
The interaction mechanisms through which HFD promotes PP by modulation of GM and its metabolic products. HFD, a high-fat diet; SCFAs, short-chain fatty acids; LPS, lipopolysaccharide; GABA, Gamma-aminobutyric acid; DA, dopamine; 5-HT, 5-hydroxytryptamine; GLP-1, glucagon-like peptide-1; BAs, bile acids; E2, estradiol; iNOS, inducible nitric oxide synthase; NO, nitric oxide; GnRH, gonadotropin-releasing hormone; LH, luteinizing hormone. Figure created with BioRender.com.

From a physiological standpoint, a pivotal connection between a HFD and PP lies in the GM, which acts as a “virtual endocrine organ” with endocrine function, and the bioactive metabolites it produces influence the host’s physiological processes. Figure 2 illustrates the interaction mechanisms through which HFD promotes PP by the modulation of GM and its metabolic products, including SCFAs, BAs, LPS, and neurotransmitters such as *γ*-aminobutyric acid (GABA), dopamine (DA), and serotonin (5-HT), which are further detailed in subsequent sections.

### 5.1 Interaction between GM and sex hormones in HFD

Mutual interactions between GM and sex hormones have been reported, particularly in the context of HFD. The GM can directly modulate sex hormone levels. For example, *β*-glucuronidase, an enzyme secreted by *Clostridium* species prevalent in HFD, activates estrogen by deconjugation, thereby enhancing its bioavailability and absorption in the gut and peripheral tissues (Zengul, 2019). Conversely, sex hormone levels can also shape GM composition and diversity. Mueller et al. noted that premenopausal healthy women had lower *Bacteroides* and *Prevotella* abundances than age-matched men, a difference absent in postmenopausal women (Mueller et al., 2006). A parallel sexual dimorphism in the composition of rodent GM emerged concomitantly with pubertal onset and was subsequently abolished after castration (Org et al., 2016). Additionally, direct evidence of the influence of sex hormones on the GM was provided in E2-induced PP mice, where an increase in *Dubosiella*, *Faecalibaculum*, and *Bifidobacterium* was noted in response to E2 (Bo et al., 2022; For a more detailed discussion on the bidirectional interactions between GM and sex hormones, refer to the review; Calcaterra et al., 2022)

A deeper exploration of this complex interplay between GM and sex hormones will advance both mechanistic understanding of PP pathogenesis and development of microbiota-targeted therapies.

### 5.2 HFD promotes PP through metabolic intermediates

#### 5.2.1 SCFAs

SCFAs are fatty acids with fewer than six carbon atoms, primarily produced in the colon through the fermentation of dietary fibers by GM. The most common SCFAs, including acetate, propionate, and butyrate, play crucial roles in gut health and overall metabolism. HFD typically suppresses the proliferation of SCFA-producing gut bacteria while fostering pathogenic bacteria growth (Onishi et al., 2017). The link between SCFAs and PP is long established, with a metabolomic study revealing negative correlations between fecal butyrate, isovalerate, and caproate levels and early puberty onset (Yuan et al., 2023). Notably, a 2022 animal study provided the first experimental evidence that SCFAs exert protective effects against HFD-induced PP by reversing pubertal symptoms through the Kiss1-GPR54-protein kinase C (PKC)-extracellular-regulated kinase 1/2 (ERK1/2) signaling pathway (Wang et al., 2022). This suggests a promising non-invasive therapeutic strategy for preventing and ameliorating PP.

Despite these promising results, the role of SCFAs in PP is incompletely understood as 16S rRNA gene sequencing studies paradoxically revealed increased SCFA-producing bacteria in PP girls (Wang Y. et al., 2024; Huang et al., 2022; Dong et al., 2020). This discrepancy may reflect fundamental differences between experimental and physiological conditions. First, animal studies utilizing supraphysiological SCFA doses through direct administration may not accurately mirror the complex gut-brain communication mediated by microbial metabolites in humans. Second, cross-sectional human studies are unable to distinguish whether increased SCFA producers are drivers or passengers in PP. Third, physiological barriers—including intestinal absorption, hepatic metabolism, and blood–brain barrier permeability—may weaken SCFAs’ effects (Parker et al., 2020), explaining why higher SCFA-producing bacterial abundance does not necessarily prevent PP. Consequently, the dysbiosis induced by HFD, leading to altered SCFA profiles, necessitates further research within the PP context.

#### 5.2.2 BAs

BAs are vital for fat metabolism, aiding in the emulsification and absorption of dietary fats and fat-soluble vitamins in the intestine. HFD significantly boosts bile acid (BA) synthesis and secretion—particularly of hydrophobic secondary BAs—with certain gut bacteria (such as *Clostridium* and Ruminococcaceae families; Araki et al., 2005; Stenman et al., 2013; Chen et al., 2023; Jiao et al., 2017) catalyzing this process via 7α-dehydroxylation reactions (Li et al., 2021a). The relationship between BAs and puberty was first noted by Bergmann et al. in 1986, who found elevated BA saturation during female puberty, positively correlating with estrogen levels (Bergmann et al., 1986). Recent integrated 16S rRNA gene sequencing and metabolomic analyses revealed notably decreased metabolites involved in primary BA biosynthesis (such as glycocholate, cholic acid, and taurochenodeoxycholic acid) in CPP, with glycocholate potentially enhancing sex hormone absorption (Huang et al., 2023). Mechanistically, HFD-induced female PP rats showed significantly reduced GDCA levels, with glycodeoxycholic acid (GDCA) supplementation ameliorating PP symptoms through the modulation of the hypothalamic *Sirt1*/*Kiss1* signaling pathway (Wu et al., 2025). BAs may also exert diverse biological effects via receptors, such as takeda G-protein-coupled receptor 5 (TGR5) and farnesoid X receptor (FXR). As Vanden Brink et al. showed, hypothalamic TGR5 receptor activation accelerated puberty in normal female rats through kisspeptin receptor-dependent GnRH secretion (Vanden Brink et al., 2024). This ‘paradox’ may be attributable to factors such as genetic background variations, context-dependent effects (HFD versus normal diet), and non-native forms of GDCA that mediate hypothalamic signaling. Importantly, significant species differences in BA metabolism—particularly the abundance of rodent-specific hydrophilic and 6α-hydroxylated BAs—underscore the need for clinical validation when translating these animal findings to human applications (Straniero et al., 2020).

#### 5.2.3 LPS

LPS, a major component of the outer membrane of Gram-negative bacteria, acts as an endotoxin that triggers strong inflammatory responses in host organisms. Mounting evidence indicates that HFD increases the prevalence of LPS-producing bacteria (such as those in the S24-7 family, Enterobacteriaceae, and Desulfovibrionaceae), and exacerbate intestinal permeability, leading to elevated intestinal LPS levels entering the bloodstream (Stenman et al., 2013; Moreira et al., 2012; Kang et al., 2017). LPS activates toll-like receptor 4 receptors on glial cells in the brain, inducing the release of inflammatory mediators and promoting potentiates oxidative stress, which may be accentuated during puberty (Iwasa et al., 2015; Zhao et al., 2019; Schoeler and Caesar, 2019).

Additionally, LPS stimulates the expression of inducible NO synthase in various cells, increasing NO levels that are involved in regulating female reproduction. A research group found that the NO synthesis pathway is more active in girls with CPP compared to healthy controls. Further mechanistic studies showed that NO not only stimulates GnRH secretion (Araki et al., 2005; Zhao et al., 2019), but may also promote follicular development through the PI3K/protein kinase B (AKT)/FoxO3a pathway, thereby accelerating puberty onset (Han G. et al., 2022; Luo et al., 2021; Li et al., 2020).

#### 5.2.4 Neurotransmitters

Most neurotransmitters closely associated with PP are produced by the GM. HFD disrupts the intestinal microbial balance, diminishing populations of *Lactobacillus* and *Bifidobacterium*, both known to produce GABA through glutamate decarboxylase activity (Chen et al., 2023; Strandwitz, 2018). Genome-wide association studies have identified several alleles associated with the GABA signaling pathway that correlate with the age of menarche (Perry et al., 2014). GABA acts as a critical inhibitory modulator of hypothalamic GnRH neurons by engaging GABA receptors, thereby suppressing GnRH secretion (Watanabe et al., 2014). Further experiments in prepubertal rhesus monkeys demonstrated that GABA inhibits the upstream gene *Kiss1* and its expression, contributing to the delay in puberty onset (Kurian et al., 2012). Additionally, GABA reduces NO production in the hypothalamus, diminishing the stimulation of the HPG axis and the promotion of follicular development (Yang et al., 2020).

In parallel, HFD also augments gut bacteria linked to intestinal inflammation (e.g., *Bilophila*, *Desulfovibrio*, and *Escherichia*), which synthesize neurotransmitters like dopamine and 5-HT, both of which are critical regulators during puberty (Chen et al., 2023; Wang M. et al., 2024). DA activates GnRH through a cAMP-mediated mechanism, enhancing the amplitude and duration of GnRH pulses to promote the onset of puberty (Winters et al., 2014). In contrast, 5-HT exerts a biphasic effect on GnRH neurons: rapid inhibition via 5-HT1A receptors, followed by slow excitation through 5-HT2A receptors (Bhattarai et al., 2014). Despite these insights, the exact mechanisms and causal relationships between neurotransmitters and PP require more detailed exploration.

## 6 Targeting GM modulation for PP

### 6.1 Dietary and lifestyle interventions

Recent advancements in dietary interventions have demonstrated their effectiveness as a supplemental strategy for managing PP by influencing GM composition and biological activity, thereby modulating the host’s sexual development. Diets rich in vegetables and proteins have shown effectiveness in mitigating the onset and progression of PP (Gu et al., 2024). Specifically, plant-based high-fiber diets may diminish estrogen levels by inhibiting the dissociation of estrogen-binding proteins and facilitating increased estrogen excretion via feces. This reduction in circulating estrogen may delay puberty onset and slow sexual maturation (Cheng et al., 2012).

In addition to dietary modifications, clinical trials have highlighted the efficacy of certain supplements in delaying sexual development. For instance, a combination of GnRHa and pomegranate extract has proven more effective than GnRHa alone in managing idiopathic CPP (Liu and Tang, 2017). Treatment with decaffeinated green tea polyphenol in prepubertal obese girls not only improved obesity but also delayed puberty onset (Xie et al., 2021). Lifestyle interventions like intermittent fasting have yielded positive outcomes in PP management (Yu et al., 2021). Taken together, while these dietary and lifestyle interventions show promise in alleviating symptoms of PP, further research is required to identify and establish the most effective and evidence-based approaches for managing this condition.

### 6.2 Probiotics

Probiotics are live microorganisms that confer health benefits to the host when administered in adequate amounts (Sanders et al., 2019). While direct clinical studies on the effects of probiotics in children with PP are lacking, emerging animal researches evaluate potential advantages. Specific gut microbial combinations, such as a regimen predominantly containing *Bifidobacterium longum* or a blend of *Lactobacillus rhamnosus* and *Lactobacillus helveticus*, have demonstrated the potential in delaying puberty onset in female mice with PP (Yuan et al., 2023; Cowan and Richardson, 2019). Future research should prioritize large-scale, randomized controlled trials to confirm the efficacy and safety of these interventions.

### 6.3 FMT

FMT is an innovative therapeutic option in treating conditions such as *Clostridium difficile* infection (Cheng Y. W. et al., 2021), diabetes (De Groot et al., 2021), metabolic syndrome (Li et al., 2016), and autism (Kang et al., 2020). This procedure involves transplanting fecal material from a healthy donor into the patient’s gastrointestinal tract to reestablish microbial balance (Hamamah et al., 2022). There are currently only two animal experimental studies that have been reported FMT could play a critical role in puberty regulation. In both studies, researchers transplanted fecal microbiota from HFD-induced PP rats and from PP girls, and observed an accelerated puberty onset in healthy rats (Bo et al., 2022). In summary, FMT could serve as a viable future treatment for PP, although more studies are needed to fully elucidate the underlying mechanisms and safety in children (Yue and Zhang, 2024).

### 6.4 Traditional Chinese medicine (TCM)

TCM has been effectively utilized for centuries to treat various health issues, including Alzheimer’s disease (Ding et al., 2022), Parkinson’s disease (Muhammad et al., 2022), rheumatoid arthritis (Jakobsson et al., 2022), and PP (Han X. X. et al., 2022). Several TCM herbs, such as Anemarrhena (Yan et al., 2021), Phellodendron (Su et al., 2021), Berberine (Yang et al., 2022), and Poria (Shan-Shan et al., 2019), show efficacy in modulating the dysregulation of the gut microbial ecosystem. Currently, commonly prescribed formulations for PP in clinical practice include Zhibai Dihuang Wan, Dabu Yin Wan, and Shugan Zhi Yin Jiao Huo Fang. A meta-analysis also revealed integrating TCM with GnRHa therapy improved treatment outcomes compared to GnRHa alone (Lee et al., 2020). Despite these advantages, integrated treatment approaches should be approached cautiously, with careful consideration to ensure that TCM complements rather than interferes with conventional therapies.

## 7 Discussion

Given the contribution already made by the study described above, we believe that future research should focus on the following key areas:

### 7.1 The relationship between HFD, GM, and PP

Extensive observational studies have established that HFD (particularly PUFAs and animal proteins) represents a significant risk factor for PP in girls (Chen et al., 2024; Gu et al., 2024; Günther et al., 2010; Xiong et al., 2022; Du et al., 2024; Cheng et al., 2021b; Xu et al., 2022; Cheng et al., 2021a; Shahatah et al., 2021; Nguyen et al., 2020; Jansen et al., 2016; Mueller et al., 2015; Carwile et al., 2015; Mervish et al., 2013; Wiley, 2011; Rogers et al., 2010; Koo et al., 2002; Berkey et al., 2000; Maclure et al., 1991; Merzenich et al., 1993; Szamreta et al., 2020). Preclinical investigations further revealed that maternal or prepubertal HFD exposure induces PP in rodent models (Bo et al., 2022; Wang et al., 2020), with the reported potential mechanisms involving two distinct yet interconnected pathways: microbiota-independent mechanisms (detailed in Section 2), including hypothalamic microglial activation, p53 upregulation, metabolic hormone dysregulation, and others, all of which have been elucidated in multiple reviews as contributing to the activation of the HPG axis (Stathori et al., 2025; Calcaterra et al., 2023; Valsamakis et al., 2021); and microbiota-dependent pathways, whereby HFD-induced gut dysbiosis directly interferes with sex hormone metabolism while modulating key metabolic mediators (such as SCFAs, BAs, and LPS) through microbial enzymatic activities. The latter pathway highlights the mediating role of gut microbiota and their metabolites in HFD-induced PP.

Although 16S rRNA gene sequencing and metabolomics from clinical and preclinical studies have identified characteristic microbial and metabolic signatures in PP, these cross-sectional approaches cannot establish causal relationships with HFD-induced PP (Bo et al., 2022; Wang et al., 2020; Wang Y. et al., 2024; Wang L. et al., 2024; Huang et al., 2023; Huang et al., 2022; Li et al., 2021b; Dong et al., 2020; Qian et al., 2024; Yi et al., 2024; Nguyen et al., 2024; Yuan et al., 2023; Wang et al., 2022). Intervention studies employing FMT and targeted metabolite administration have confirmed that GM influences HFD-induced PP by regulating the hypothalamic Kiss1-GnRH system, with specific metabolites like SCFAs and GDCA exerting ameliorative effects through this pathway (Bo et al., 2022; Wang et al., 2022; Wu et al., 2025). GDCA upregulated *Sirt1* expression to suppress the *Kiss1* gene. Intriguingly, SCFAs (Jiao et al., 2020; Caetano-Silva et al., 2023; Lin et al., 2022) and specific BAs (Shihabudeen et al., 2015; Yanguas-Casás et al., 2017; Paluschinski et al., 2019; such as tauroursodeoxycholic acid, glycochenodeoxycholic acid, chenodeoxycholic acid, and others) may inhibit multiple key nodes in microbiota-independent pathways, including attenuating p53 expression, reducing microglial activation, and modulating metabolic hormone dysregulation. Leptin, in turn, has been reported to modulate gut microbial metabolic function by enhancing duodenal sympathetic tone and to regulate systemic BA metabolism (Wen et al., 2021; Toledo et al., 2025). However, critical issues still remain: first, the discordance between increased SCFA-producing bacteria and their biological effects in PP may stem from multiple factors, such as pharmacologic versus physiologic concentration disparities, cross-sectional study limitations, and distinct mechanisms of direct interventions versus microbial-mediated actions; second, species-specific variations in BA metabolism constrain clinical translation to humans (Straniero et al., 2020); third, it remains unclear the specific microbial sources of bioactive metabolites and how GM and metabolites regulate hypothalamic gene expression via the microbiota-gut-brain (MGB), potentially through vagal or circulatory pathways (detailed in Section 7.2).

Additionally, key neuromodulators (GABA, DA, 5-HT) and LPS are promising metabolites warranting further investigation, though their precise roles and concentration changes in HFD-induced PP remain to be elucidated. Collectively, HFD impacts pubertal timing through both microbiota-dependent and microbiota-independent pathways that exhibit cross-regulation, providing a conceptual framework for understanding PP pathogenesis and developing targeted interventions.

### 7.2 Evidence between the gut-brain axis and PP

Mounting evidence linking the GM to hypothalamic dysfunction in the context of PP has underscored the bidirectional communication between the GM and the brain via the MGB axis. MGB axis is theorized to occur through various systems, including the autonomic and enteric nervous systems, neuroendocrine pathways, and the immune system (Cryan et al., 2019). Nowadays, understanding of the mechanisms of PP through the MGB axis focuses on several pathways.

First, GM may modulate brain function through vagal nerve activation, the most extensively studied pathway. Clinical evidence shows cardiac autonomic dysfunction in girls with idiopathic PP, mainly manifested as a decrease in vagal nerve tension (Yi et al., 2017). Animal experiments further demonstrate that prepubertal vagotomy could lead to ovarian dysfunction, confirming its crucial role in the development of puberty (Morales-Ledesma et al., 2004). Mechanistically, as the main conduction pathway connecting the enteric nervous system and the central nervous system, the vagus nerve expresses receptors for a variety of bioactive compounds on its surface (such as GLP-1 receptor, cholecystokinin 1 receptor, neuropeptide Y receptor Y2, GPR35, and GPR119), enabling direct recognition of signaling molecules from GM and their metabolites, such as LPS-producing bacteria (Ma et al., 2024), acetate-producing bacteria and acetate (Zheng et al., 2021), kynurenic acid (Fan et al., 2023b), indole (Jaglin et al., 2018) and others. While cross-disease studies provide experimental support for vagus nerve–mediated microbiota-brain communication, critical gaps persist in understanding its specific regulatory mechanisms in PP pathogenesis—particularly how distinct microbial metabolites modulate HPG axis activity via the vagus nerve.

Second, under pathological conditions, harmful gut microbes and their metabolites in the bloodstream can also disrupt hypothalamus function. Given its anatomical proximity to the blood–brain barrier (BBB), the hypothalamus is particularly vulnerable to the detrimental effects of BBB dysfunction and leakage (Thaler et al., 2012). Developmental studies have shown that sex hormones in the neonatal period can significantly regulate the development of cerebral blood vessels and the integrity of the BBB. This critical period coincides with the early activation peak of the HPG axis, providing important clues for understanding the association between PP and abnormal BBB function (Collignon et al., 2024). In addition, a study demonstrated a causal relationship between microbial alterations and gut barrier dysfunction, as alterations were observed in gut barrier permeability and in tight junction protein occludin in both the frontal cortex and hippocampus in germ-free adult mice, and these alterations were shown to recover following treatment with SCFA-producing bacterial strains or sodium butyrate (Braniste et al., 2014). These findings provide direct evidence for the regulation of neuroendocrine function by the MGB axis, suggesting that the GM may play a crucial role in the pathogenesis of PP by affecting the integrity of the BBB. However, direct animal and clinical data on the specific regulatory mechanisms of the MGB axis in PP are remain lacking, underscoring the urgent need for future research in this field.

Although there is growing discussion about the role of the MGB axis in regulating neurological and metabolic disorders, direct animal and clinical evidence in the context of PP remains lacking. Future research in this area is urgently needed.

### 7.3 The role of HFD and obesity

The relationship between HFD, obesity, and PP has been a subject of ongoing debate. Emerging evidence confirms that HFD can indeed trigger PP through obesity-independent pathways: first, animal studies demonstrate that both HFD-induced and kisspeptin-induced PP occur independently of weight changes, with short-term HFD exposure sufficient to elevate gonadotropins without altering body fat or leptin levels (Ullah et al., 2019; Frisch et al., 1977; Sahin et al., 2015); second, gut microbes specifically enriched in female rats with PP (such as *Bilophila*; Kimura et al., 2013; Kimura et al., 2011), *Lachnoclostridium* (Zhao et al., 2021), *Lactobacillus* (Henning et al., 2018), *Lactobacillus murinus* (Li et al., 2024), *Lactobacillus reuteri* (Zhang et al., 2022) are negatively correlated with obesity, indicating differences between the microbial communities regulating pubertal development and the microbiota associated with obesity; third, the conventional paradigm recognizes obesity as a significant risk factor for PP through its induction of metabolic hormone dysregulation, a well-established mechanism extensively documented in reviews (Shi et al., 2022; Calcaterra et al., 2023). However, HFD serves as the primary instigator of both metabolic hormone imbalance and subsequent obesity development (Chakraborty et al., 2016; Fan et al., 2023a). Under HFD conditions, metabolic hormone dysregulation and obesity exhibit complex bidirectional interactions, with obesity serving as an exacerbating factor rather than a prerequisite for metabolic dysfunction. This is evidenced by findings that long-term HFD-fed mice exhibit increased GLP-1 secretion and insulin resistance independent of obesity and body weight (Wang et al., 2015; Clegg et al., 2011), and girls with CPP show high serum leptin levels uncorrelated with body mass index (BMI; Zurita-Cruz et al., 2021; Kang et al., 2018). Collectively, these findings confirm that HFD induces PP not only through the traditional “obesity-metabolic hormone” pathway but also via obesity-independent mechanisms.

### 7.4 Integrated omics technologies and population research

Intestinal microbes and their metabolic byproducts provide new biological insights into the link between HFD and PP. However, existing research still has significant limitations. Firstly, the current evidence primarily relies on cross-sectional designs and the low-resolution 16S rRNA gene sequencing technology, which limits the comprehensive revelation of dynamic evolution of GM and lacks precision at the species level. To break through this bottleneck, future research should prioritize conducting longitudinal cohort studies combined with high-throughput technologies such as metagenomic sequencing for multi-omics integration analysis (including metabolomics, epigenomics, and proteomics). Such research can dynamically capture the key biological processes in the occurrence and development of PP, identify reliable biomarker prediction models through machine learning algorithms, and deeply analyze the “microorganisms-metabolites-hosts” interaction network. The feasibility of this methodological approach has been well established in prior research. For example, Huang et al. developed a diagnostic classifier to differentiate CPP from healthy controls using integrated microbiome-metabolome analysis, identifying potential therapeutic biomarkers (Huang et al., 2023). In addition, complementary animal studies using combined transcriptomic-epigenomic profiling elucidated critical regulatory genes and pathway networks in PP pathogenesis (Mohamed et al., 2022). Secondly, most mechanistic data on PP is largely based on animal experiments and lacks sufficient clinical evidence to definitively support its role in humans. Given the complexity and specificity of the disease, large-scale clinical studies are likely to provide more valuable insights into its pathophysiology than animal models.

In conclusion, HFD-induced PP arises from the overlapping effects of GM dysbiosis and high-fat intake, involving multiple mechanisms. Fortunately, advances in omics research and breakthroughs in mechanistic studies using animal models are rapidly enhancing our understanding of the causal mechanisms between HFD, GM, and PP. These advancements also reveal the potential therapeutic effects of key bacterial species or taxa, paving the way for optimizing microbiota-based therapeutic strategies and developing innovative, non-invasive treatment options for children with PP.

## Glossary

PP: precocious puberty
HFD: a high-fat diet
GM: gut microbiota
CPP: central precocious puberty
PPP: peripheral precocious puberty
HPG: hypothalamic–pituitary-gonadal
NO: nitric oxide
E2: estradiol
LPS: lipopolysaccharides
SCFAs: short-chain fatty acids
SCFA: short-chain fatty acid
BA: bile acid
BAs: bile acids
GABA: γ-aminobutyric acid
5-HT: serotonin
DA: dopamine
GLP-1: glucagon-like peptide-1
GPR: G-protein coupled receptor
FMT: Fecal Microbiota Transplantation
TCM: Traditional Chinese medicine
MGB: microbiota-gut-brain
BBB: blood–brain barrier
GDCA: glycodeoxycholic acid
BMI: body mass index
PKC: protein kinase C
ERK1/2: extracellular signal-regulated kinase1/2
LH: luteinizing hormone
FSH: follicle-stimulating hormone
APN: adiponectin
IGF-1: insulin-like growth factor-1
PI3K: phosphatidylinositol-3-kinase
MUFAs: monounsaturated fatty acids
PUFAs: polyunsaturated fatty acids
TGR5: takeda G-protein-coupled receptor 5
FXR: farnesoid X receptor
mTOR: mammalian target of rapamycin
SF1: steroidogenic factor 1
Mdm2: mouse double minute 2 homolog
AMPK: adenosine-monophosphate-activated protein kinase
ACTH: adrenocorticotropic hormone
ERα: estrogen receptor α
GHSR: growth hormone secretagogue receptors
AKT: protein kinase B
