# Supplementary material for: Relationship between high-fat diet, gut microbiota, and precocious puberty: mechanisms and implications
Source: Front Microbiol. 2025 Jun 4;16:1564902. doi: 10.3389/fmicb.2025.1564902 (PMC12174423; doi:10.3389/fmicb.2025.1564902)
Supplement: Supplementary file 1 [file Table_1.docx]

Supplementary Material

# Supplementary Tables

**Supplementary Table 1.** **Epidemiological evidence on the relationship between HFD and pubertal timing.**

| Study Design | Sample | Main Findings | Year | |
| --- | --- | --- | --- | --- |
| Cross-sectional study | 4085 Chinese children aged 6–9 years | Compared to the Westernized dietary pattern, children who predominated the Traditional dietary pattern were protectively associated with PP. | 2024^[5]^ | |
| Case-control study | 185 PP Chinese girls and 185 age-matched controls | 1. The high animal food and fruits dietary pattern, and higher red meat consumption were strongly associated with an increased risk of PP.  2. The high vegetable and protein dietary pattern was significantly negatively associated with PP. | 2024^[6]^ | |
| Case-control study | 424 Chinese girls aged 4.5–9.3 years | Girls with breast development and central priming tended to consume fewer vegetables and less milk than healthy girls without breast development. | 2024^[9]^ | |
| Prospective study | 4781 Chinese children (2152 girls and 2629 boys) aged 6–8 years | Higher soy intake was associated with later puberty timing in girls and boys. | 2022^[8]^ | |
| Data collected from the ALSPAC | 7730 children (3919 girls and 3811 boys) | Higher protein intake was associated with earlier breast development, peak height velocity, and menarche in girls. | 2022^[10]^ | |
| Data collected from the CHNS and SCCNG Study | 5920 Chinese children (3425 girls and 2495 boys) | A higher intake of dietary fat and PUFAs in prepuberty was associated with earlier puberty timing, which was independent of dietary protein intake. | 2022^[11]^ | |
| Data collected from the ALSPAC | 7526 British children (3872 girls and 3654 boys) at 8–17 years | Elevated plasma levels of dihomo-γ-linolenic acid, an intermediate metabolite of n-6 PUFAs, contributed to the onset of early puberty in girls. | | 2021^[12]^ |
| Cross-sectional study | 164 Saudi females aged 5–20 years | 1. A significant correlation between higher weight standard deviation and an earlier age of both thelarche and adrenarche was found.  2. Consumption of fast foods was significantly associated with an earlier age at menarche.  3. Consumption of non-organic poultry was linked to early thelarche. | | 2021^[13]^ |
| Longitudinal study | 202 girls from New Jersey aged 9-10 years at baseline (2006–2014) | Girls with higher adherence to a Mediterranean-like diet had a later age at menarche and thelarche compared to those with lower adherence. | | 2020^[25]^ |
| Systematic review and meta-analysis | 10884 girls | 1. The intake of PUFAs was associated with an increased risk of earlier menarche, with a dose-response effect.  2. For each additional 1 g/day animal protein intake in childhood, the age at menarche was approximately two months earlier.  3. Girls with a high intake of fiber and MUFAs in childhood experienced later menarche onset. | | 2020^[14]^ |
| Prospective study | 456 girls from Bogotá aged 8.4 ± 1.7 years, followed for a median 5.6 years | 1. Girls who consumed red meat at least 2 times/day reached menarche about 0.4 years earlier than their peers with a lower intake.  2. Girls with tuna/sardine intake more than 1 time/week had a significantly later age at menarche than those with intake less than 1 time/month. | | 2016^[15]^ |
| Prospective study | 2379 girls (1213 African American, 1166 Caucasian) aged 9–10 years and followed them for 10 years | 1. The intakes of caffeinated and artificially sweetened soft drinks were positively associated with risk of early menarche.  2. Greater consumption of caffeine and aspartame was associated with a higher risk of early menarche. | | 2015^[16]^ |
| Prospective study | 5583 American girls aged 9–14 years and followed them for 6 years | Milk, total meat, and red meat consumption were not related to the age at onset of menarche. | | 2015^[17]^ |
| Longitudinal study | 1178 American girls aged 6–8 years | Girls with a flavonoid intake of at least 5 mg/day experienced later breast development compared to those with an intake of no more than 2 mg/day. | | 2013^[18]^ |
| Data collected from the NHANES 1999-2004 | 1008 American girls aged 9–12 years | Higher milk intake is linked to an increased risk of early menarche or a younger age at menarche. | | 2011^[19]^ |
| Data collected from the DONALD | 112 German children (57 girls and 55 boys) aged 6–13 years | Dietary fat intake is positively correlated with earlier onset of pubertal growth and peak height velocity. | | 2010^[7]^ |
| Data collected from the ALSPAC | 3298 British girls aged 3–10 years | Higher total and animal protein intake, along with PUFAs and meat consumption at ages 3 and 7 years promoted early menarche. | | 2010^[20]^ |
| Prospective cohort study | 637 Canadian girls aged 6–14 years | A higher intake of energy-adjusted dietary fiber was associated with a lower risk of menarche. | | 2002^[21]^ |
| Longitudinal study | 67 Caucasian girls, birth to 10 years of age | 1. Girls who consumed more animal protein and less vegetable protein at ages 3–5 years experienced earlier menarche 2. Girls aged 1–2 years with higher dietary fat intakes and girls aged 6–8 years with higher animal protein intakes became adolescents with earlier peak growth. | | 2000^[22]^ |
| Prospective study | 261 American girls aged 8–15 years | Higher fat intake was associated with accelerated menarche, whereas physical activity exhibited the opposite effect. | | 1993^[24]^ |
| Prospective study | 213 girls aged 10 years | Menarcheal age was not associated with the intake of energy or energy-adjusted intake of protein, fat, or carbohydrate. | | 1991^[23]^ |

Abbreviations: PP, precocious puberty; PUFAs, polyunsaturated fatty acids; n-6 PUFAs, omega-6 polyunsaturated fatty acids; MUFAs, monosaturated fatty acids; ALSPAC, Avon Longitudinal Study of Parents and Children; CHNS, China Health and Nutrition Survey; SCCNG, Southwest China Childhood Nutrition and Growth; NHANES, National Health and Nutrition Examination Survey; DONALD, Dortmund Nutritional and Anthropometric Longitudinally Designed Study.

**Supplementary Table 2. The association between GM and PP.**

| Model | Sample Details | Method | Microbial composition alteration | Metabolites and other alterations | Year |
| --- | --- | --- | --- | --- | --- |
| Human | CPP (N = 21) PPP (N = 45) Healthy girls (N = 48) | 16S rRNA sequencing, untargeted metabolomics sequencing | 1. The PP group (CPP and PPP) had higher levels of SCFAs-producing bacteria including *Tyzzerella*, *Butyricicoccus*, *Ruminococcus*, and *Erysipelatoclostridium ramosum*. 2. PP-enriched *Bacteroides* was associated with lower fruit intake and higher levels of LH and FSH. | The CPP and PPP groups had higher levels of lipoperoxidation products, leading to increased oxidative stress. | 2024^[74]^ |
| Human | Healthy girls (CTR, N = 41) Normal-weight PP (PP, N = 42) Obesity-related PP (OPP, N = 42) | 16S rRNA sequencing | 1. The PP and OPP groups exhibited lower levels of *Bifidobacterium*, *Bacteroides*, *Anaerostipes* and *Fusicatenibacter*. 2. Microbial biomarkers distinguishing the CTR and PP groups included *Acinetobacter*, *Clostridium* *innocuum*, *Glutamicibacter*, *Prevotella 7* and *Aquabacterium.* 3. In the PP and OPP groups, lower levels of *Anaerostipes* were negatively correlated with body weight, BMI, bone age, LH, FSH, and E2. |  | 2024^[75]^ |
| Human | CPP (N = 91)  Healthy girls (N = 59) | 16S rRNA sequencing, untargeted metabolomics sequencing | The CPP group exhibited higher levels of *Bifidobacterium*, *Blautia*, and *Streptococcus*. | 1. The CPP group exhibited higher activity in pathways related to isovaleric acid synthesis, propionic acid degradation, and NO synthesis. 2. The CPP group showed reduced levels of blood metabolites, including glycochenodeoxycholate, glycocholate, trihydroxycoprostane, cholic acid, and taurochenodeoxycholic acid. | 2023^[76]^ |
| Human | ICPP (N = 27) PPP (N = 18) Healthy girls (HC, N = 23) | 16S rRNA sequencing | The ICPP and PPP groups had higher levels of butyrate-producing bacteria *Prevotella*, *Roseburia*, *Ruminococcus*, and *Alistipes*, while *Bacteroides* and *Faecalibacterium* showed an opposite trend, with *Bacteroides* gradually decreasing from HC to PPP, and then to the ICPP group. | The ICPP girls exhibited enriched pathways related to cell motility, signal transduction, and environmental adaptation, while the carbohydrate metabolism pathway was less abundant. | 2022^[77]^ |
| Human | CPP (N = 27)  Overweighted girls (N = 24) Healthy girls (N = 22) | 16S rRNA sequencing | 1. The CPP girls exhibited overrepresented *Alistipes*, *Klebsiella*, and *Sutterella*, linked to neuroendocrine functions. 2. CPP-enriched *Parabacteroides* positively correlated with LH, while serotonin-producer *Akkermansia* exhibited negative relationships with FSH and LH. | The CPP girls presented higher levels of the neuroendocrine-related pathways, including acetate synthesis, DA synthesis and NO synthesis. | 2021^[78]^ |
| Human | ICPP (N = 25)  Healthy girls (N = 23) | 16S rRNA sequencing | 1. The ICPP group exhibited increased GM species linked to obesity and SCFAs production, including *Ruminococcus gnavus*, *Ruminococcus callidus*, *Ruminococcus bromii*, *Roseburia inulinivorans*, *Coprococcus eutactus*, *Clostridium leptum*, and *Clostridium lactatifermentans*. 2. Higher abundances of *Fusobacterium* and *Gemmiger* in the ICPP group were positively correlated with FSH and LH, respectively. | The ICPP girls exhibited enriched pathways related to cell motility, signal transduction, and environmental adaptation. | 2020^[79]^ |
| Human and Female Sprague-Dawley rat | Human: Healthy girls (N = 10); Normal-weight PP (N = 10); Obesity-related PP (N = 10) Rat: FMT from obese precocious girls to female recipient rats | 16s rRNA sequencing | 1. *Bifidobacterium*, *Collinsella*, and *Romboutsia* were inversely associated with obesity-related PP, while *Dialister* and *Bacteroides* showed the opposite result. 2. FMT led to an earlier puberty onset, accompanied by increased serum levels of LH, FSH, and E2, as well as elevated hypothalamic expression of *Kiss1* and *GnRH* genes. |  | 2024^[80]^ |
| Female Sprague-Dawley rat | ICPP model rats were established by a single subcutaneous injection of Danazol on PND 5 (N = 12). | 16S rRNA sequencing | ICPP downregulated the abundance of *Prevotella* and increased the abundance of *Bifidobacterium*, *Muribaculaceae*, and *Lactobacillus*. |  | 2024^[81]^ |
| Sprague–Dawley rats and C57BL/6 mice | PP model rats were induced with a single subcutaneous injection of Danazol on PND 5 (N = 6). | 16S rRNA sequencing, targeted SCFAs sequencing | PP model rats showed increased relative abundances of several Lachnospiraceae species, but decreased relative abundances of some Lachnoclostridium species. | PP model rats exhibited elevated fecal levels of isobutyric acid. | 2024^[82]^ |
| Female C57BL/6 mice | DAI gavaged from PND 21 to PND 27 induced early puberty (N = 9). | 16S rRNA sequencing, targeted SCFAs sequencing | DAI induced early puberty was associated with a higher prevalence of *Enterococcus*, *Christensenella*, and *Akkermansia*, while *Prevotella*, *Sutterella*, *Oscillospira*, *Dehalobacterium*, *Coprobacillus*, *Anaerotruncus*, and *Mucispirillum* were less prevalent. | DAI-induced early puberty was associated with lower butyric acid levels. | 2023^[83]^ |
| Female C57BL/6 mice | The PP model was constructed by feeding HFD from PND 21 to PND 35, performing FMT, and injecting E2, respectively (N = 5–8). | 16S rRNA sequencing | 1. In HFD-induced PP, GnRH was positively correlated with *Desulfovibrio*, *Lachnoclostridium*, *Streptococcus*, *Anaerotruncus*, and *Bifidobacterium*. 2. Fecal microbiota transplants from HFD-induced PP rats advanced puberty onset in healthy rats, accompanied by upregulation of GnRH and Kiss1 expression in the hypothalamus. 3. Estrogen changed the composition and proportion of GM and promoted PP. | HFD-induced PP increased serum E2, leptin, and DCA. | 2022^[26]^ |
| Female Sprague-Dawley rat | The PP model was induced by feeding with HFD from PND 21 to PND 42 (N = 6–8). | 16S rRNA sequencing, targeted SCFAs sequencing | PP model rats showed a decrease in *Lactobacillus* and an increase in *Chlamydia*. | PP model rats had significantly lower levels of acetic, propionic and hexanoic acids, with increased expression of GPR43, GPR41 and GPR109a in the colon. | 2022^[84]^ |
| Female C57BL/6 mice | Maternal HFD during lactation caused early puberty in female offspring (N = 12). | 16S rRNA sequencing | Co-housing offspring from maternal HFD during lactation with those from a maternal standard diet reversed the PP. |  | 2020^[27]^ |

Abbreviations: ICPP, idiopathic central precocious puberty; CPP, central precocious puberty; PPP, peripheral precocious puberty; PND, postnatal day; HFD, a high-fat diet; DAI, Daidzein; DCA, deoxycholic acid; ZBDH, Zhibai dihuang pill; SCFAs, short-chain fatty acids; GM, gut microbiota; FSH, follicle-stimulating hormone; LH, luteinizing hormone; HC, Healthy Control; BMI, Body Mass Index; E2, Estradiol; GnRH, gonadotropin-releasing hormone; GPR, G-protein Coupled Receptor; NO, nitric oxide; FMT, Fecal microbiota transplants; DA, dopamine.
